# Supplementary material for: BCAS2 is involved in alternative mRNA splicing in spermatogonia and the transition to meiosis
Source: Nat Commun. 2017 Jan 27;8:14182. doi: 10.1038/ncomms14182 (PMC5290162; doi:10.1038/ncomms14182)
Supplement: Supplementary Information — Supplementary Figures and Supplementary Tables [file ncomms14182-s1.pdf]

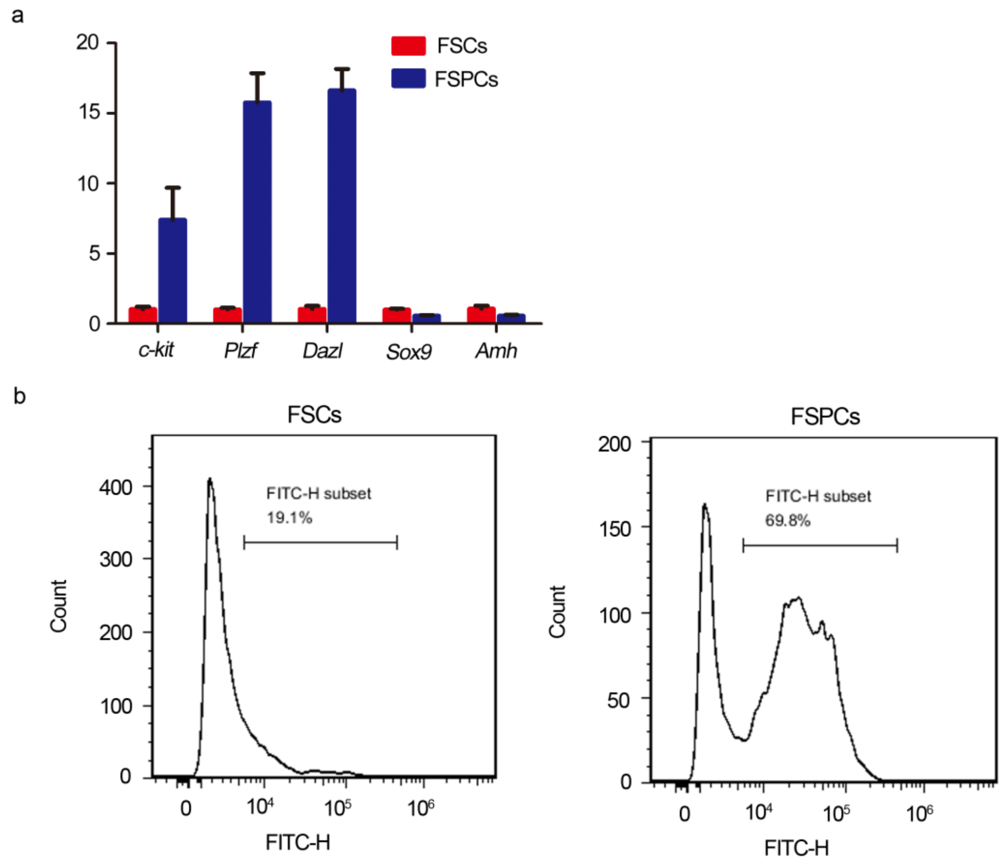

Supplementary Fig 1. Analysis the enrichment of spermatogenic cells and somatic cells. (a) Real-time RT-RCR analyzed the expression of markers in the fraction of spermatogenic cells (FSPCs) and the fraction of somatic cells (FSCs) enriched from the testes of P9 mice with *Gapdh* as internal control. *Plzf*, *c-Kit* and *Dazl* were used as the germ cells markers, meanwhile, *Sox9* and *Amh* were used as the somatic cells markers. Error bars represent s.e.m. (b) Flow cytometry analysis the ratio of MVH positive cells in FSPCs and FSCs enriched from P9 testes.

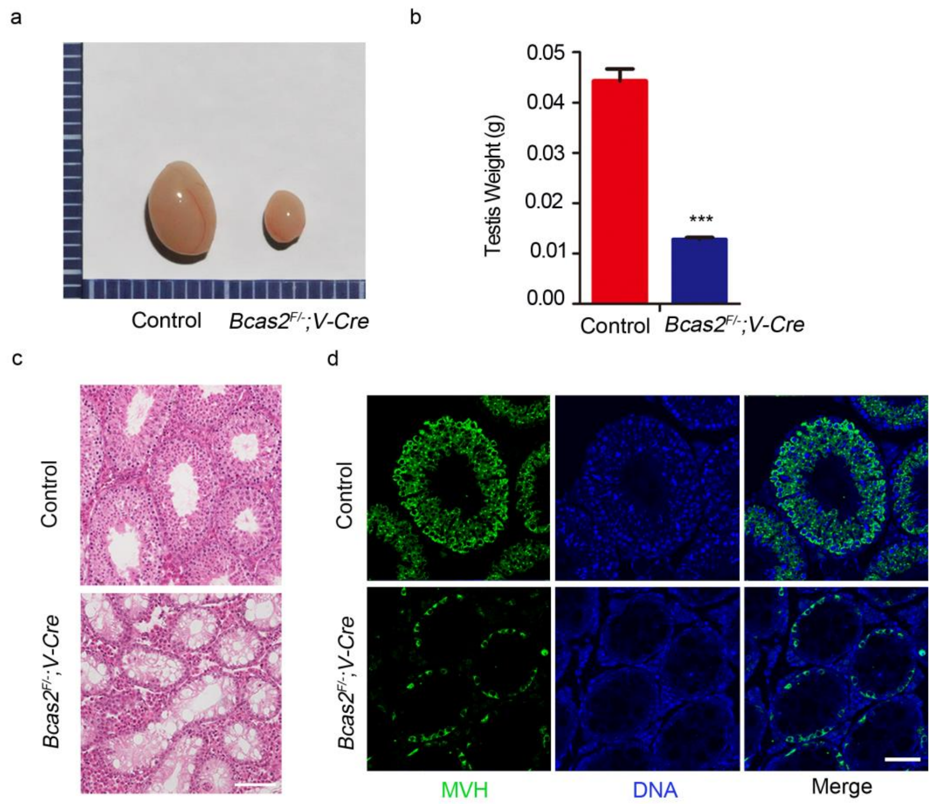

Supplementary Fig 2. BCAS2 is required for spermatogenesis in one-month-old testes. (a) Morphological analysis of one-month-old testes showed that the *Bcas2*$^{F/-}$*;Vasa-Cre* testes were smaller than the control. (b) Testes weight of one-month-old control and *Bcas2*$^{F/-}$*;Vasa-Cre* mice (****P*<0.001, n=5). Error bars represent s.e.m. (c) Hematoxylin and eosin (H&E) staining of control and *Bcas2*$^{F/-}$*;Vasa-Cre* testes in one-month-old mice. Spermatocytes and spermatids were almost absent in the seminiferous tubules of the *Bcas2*$^{F/-}$*;Vasa-Cre* mice. Scale bar, 100 μm. (d) IF staining of MVH (a germ cell marker) in control and *Bcas2*$^{F/-}$*;Vasa-Cre* one-month-old old mice. Compared to the control, only a few MVH positive cells in the basement membrane were observed in *Bcas2*$^{F/-}$*;Vasa-Cre* testes. The DNA was stained with Hoechst 33342. Scale bar, 50 μm.

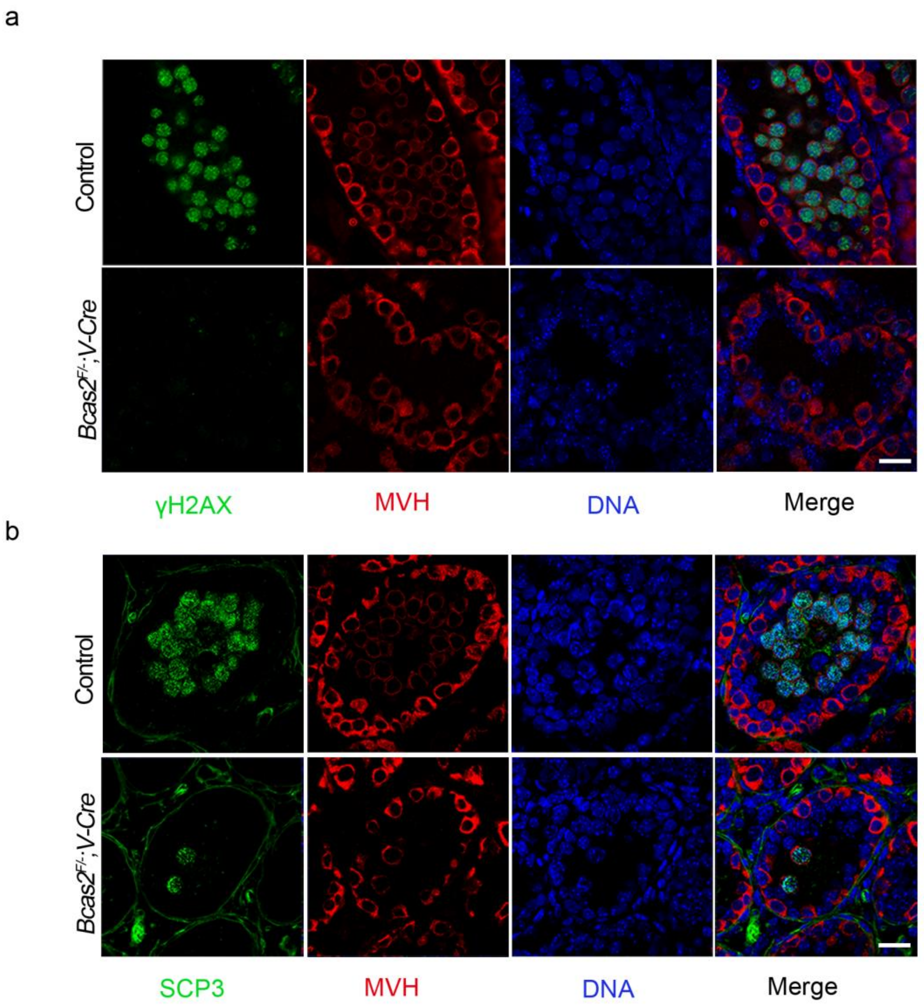

Supplementary Fig 3. Deficiency of meiosis event in prophase I of *Bcas2$^{F/-}$;Vasa-Cre* testes. (a) IF staining for SCP3 of control and *Bcas2$^{F/-}$;Vasa-Cre* testes at P12. MVH was used as a germ cell indicator. The DNA was stained with Hoechst 33342. Scale bar, 20 μm. (b) IF staining for γH2AX in control and *Bcas2$^{F/-}$;Vasa-Cre* testes at P12. MVH was used as a germ cell indicator. The DNA was stained with Hoechst 33342. Scale bar, 20 μm.

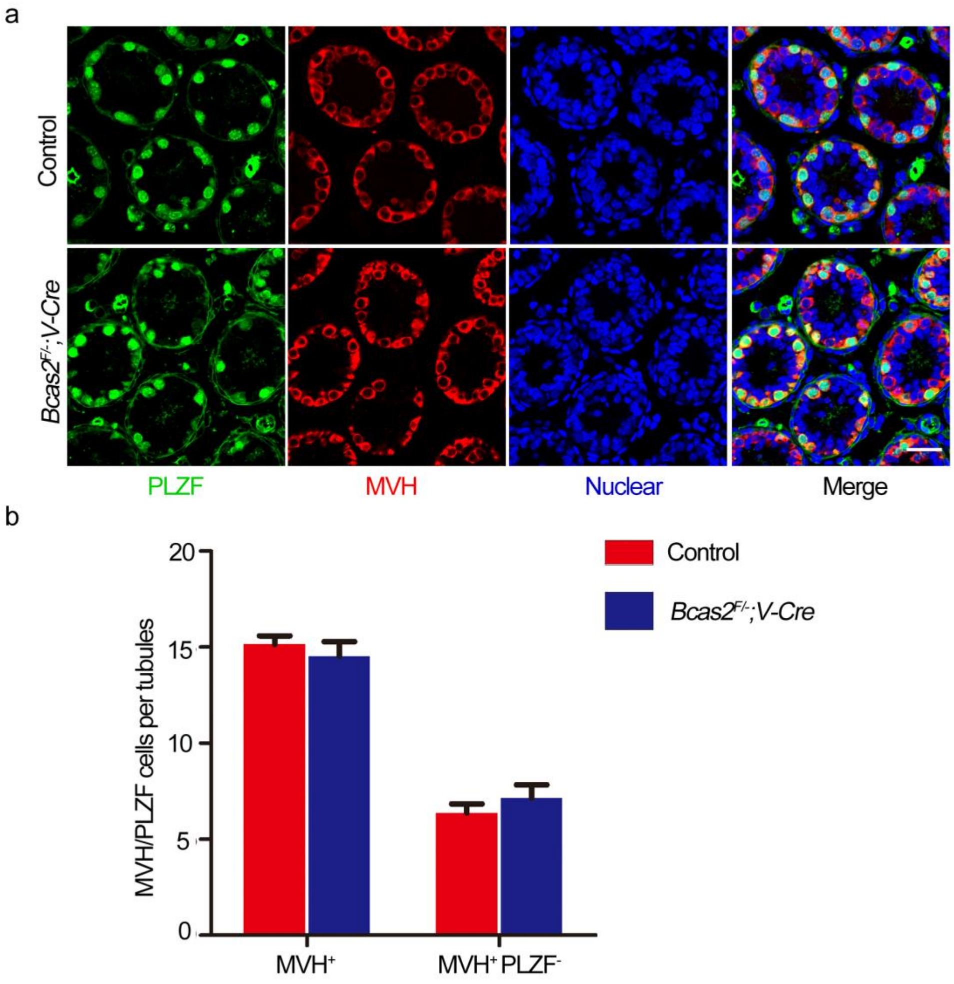

Supplementary Fig 4. Analysis of development stages of spermatogonia in *Bcas2$^{F/-}$;Vasa-Cre* testes. (a) Double staining of PLZF (Green) and MVH (Red) in sections of control and *Bcas2$^{F/-}$;Vasa-Cre* testes at P10. Scale bar, 25 μm. (b) The number (mean±s.e.m.) of MVH^{+} and PLZF^{-}MVH^{+} cells per seminiferous tubules cross-section in control and *Bcas2$^{F/-}$;Vasa-Cre* testes at P10. At least 200 tubules were counted from 3 independent mice. Error bars represent s.e.m.

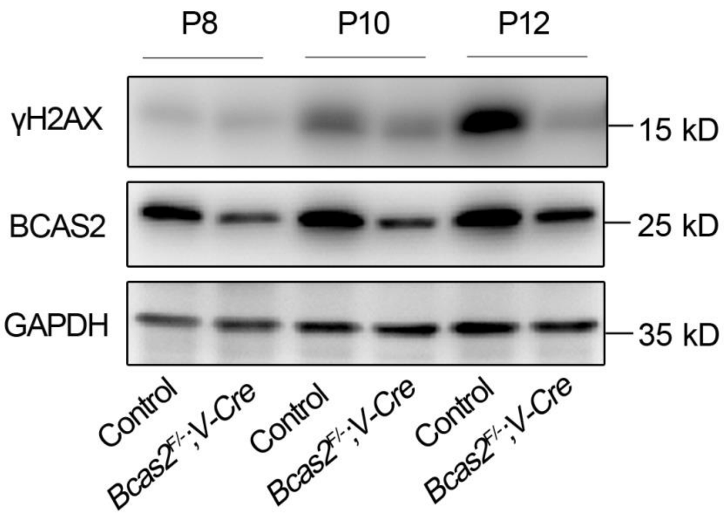

Supplementary Fig 5. DNA damage signals do not accumulate in *Bcas2$^{F/-}$;Vasa-Cre* testes during the transition of mitosis to meiosis. Western analysis the expression of γH2AX which is marker of double-stranded DNA damage (DSB) in DNA damage in control and *Bcas2$^{F/-}$;Vasa-Cre* testes at P8, P10 and P12. The level of BCAS2 protein showed the samples of control and *Bcas2$^{F/-}$;Vasa-Cre* mice. GAPDH was used as loading control.

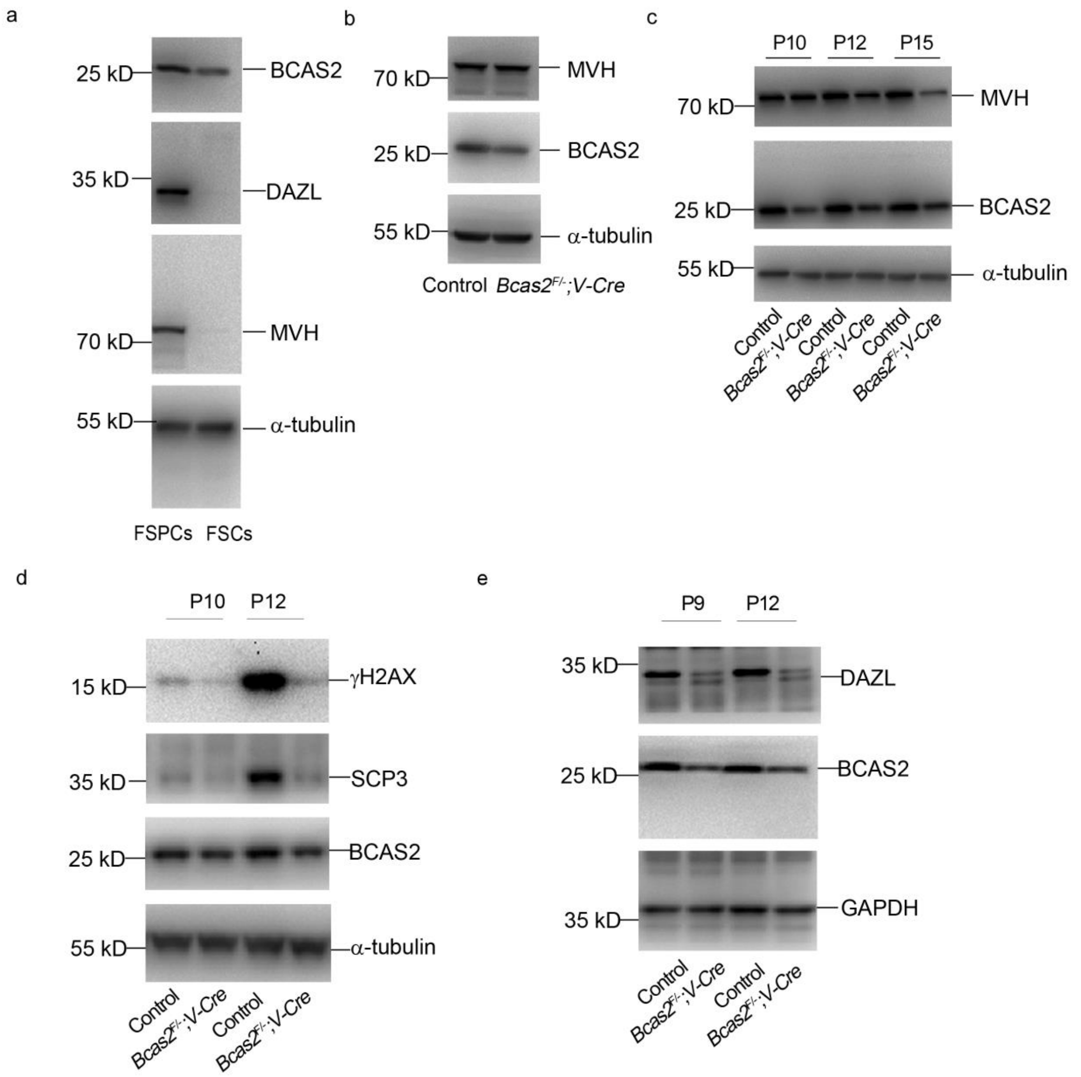

Supplementary Fig 6. Original gels shown in the main manuscript. (a) Blots correspond to shown in Figure 1d. (b) Blots correspond to shown in Figure 2b. (c) Blots correspond to shown in Figure 3a. (d) Blots correspond to shown in Figure 3c. (e) Blots correspond to shown in Figure 6d.

Supplementary Table 1. Summary of RNA-seq data mapping results

Sample name	Control	*Bcas2$^{F/-}$;V-Cre*
Total reads	51803114	51446816
Clean reads	50610844 (100%)	49955178 (100%)
Total mapped	46333767 (91.55%)	45740076 (91.56%)
Multiple mapped	1170662 (2.31%)	1119594 (2.24%)
Uniquely mapped	45163105 (89.24%)	44620482 (89.32%)

Supplementary Table 2. A list of 7 significantly changed transcriptions

Gene ID	Gene Symbol	RPKM-Control	RPKM-*Bcas2*$^{F/-}$*;V-cre*	Fold Change	P-value
ENSMUSG00000003545	FosB	10.92146	0.2758	39.59750	0.001992536
ENSMUSG00000006574	Slc4a1	15.68347	0.60653	25.85761	7.55E-05
ENSMUSG00000056054	S100a8	9.34920	0.659802	14.16969	0.003811089
ENSMUSG00000056071	S100a9	9.82674	0.400996	24.5058	0.003811089
ENSMUSG00000080893	Gm15920	24.3526	7.720678	3.154213	0.002910623
ENSMUSG00000081824	BC002163	39.33253	13.71876	2.86706	0.000396505
ENSMUSG00000095597	Gm6472	29.10597	9.837943	2.95854	0.001501834

Fold Change: RPKM-Control/ RPKM-*Bcas2*$^{F/-}$*;V-Cre*.

Fold Change>2 as the threshold for significantly differential expression

P-value: Corrected P-value <0.005 as the threshold for significantly differential expression

on

Supplementary Table 3. A list of significantly changed transcriptions in P9 testes

Down-regulated in *Bcas2*$^{F/-}$*;V-cre* sample

GeneNames	Gene Symbol	RPKM-control	RPKM-*Bcas2*$^{F/-}$*;V-cre*	FC-normalized	p-value
ENSMUSG00000060636	RpL35A	711.99	472.22	0.66	3.35E-11
ENSMUSG00000067702	Tuba3a	237.21	131.77	0.56	8.22E-08
ENSMUSG00000036752	Tubb4b	431.96	282.78	0.65	9.74E-08
ENSMUSG00000006574	SLC4A1	15.68	0.61	0.04	7.55E-05
ENSMUSG00000081824	BC002163	39.33	13.72	0.35	0.000396505
ENSMUSG00000067338	Tuba3b	136.44	83.09	0.61	0.000587107
ENSMUSG00000028393	Alad	146.90	91.69	0.62	0.000620042
ENSMUSG00000057933	gstA2	89.59	50.67	0.57	0.001421674
ENSMUSG00000095597	Gm6472	29.11	9.84	0.34	0.001501834
ENSMUSG00000031431	TSC22D3	97.82	57.12	0.58	0.001943679
ENSMUSG00000003545	fosB	10.92	0.28	0.03	0.001992536
ENSMUSG00000080893	Gm15920	24.35	7.72	0.32	0.002910623
ENSMUSG00000056071	S100A9	9.83	0.40	0.04	0.003811089
ENSMUSG00000056054	S100A8	9.35	0.66	0.07	0.003811089
ENSMUSG00000094777	Hist1h2ap	91.41	56.51	0.62	0.005752131
ENSMUSG00000061232	H2-K1	59.02	32.29	0.55	0.006407903
ENSMUSG00000042462	DCTPP1	84.96	51.58	0.61	0.006546874
ENSMUSG00000073940	Hbb-bt	42.42	20.31	0.48	0.00692075
ENSMUSG00000029848	STRA8	87.25	56.82	0.65	0.013635895
ENSMUSG00000065037	Rn7sk	20.02	7.97	0.40	0.01613516
ENSMUSG00000032291	CRABP1	76.06	48.05	0.63	0.016609513
ENSMUSG00000073411	H2-D1	59.31	35.48	0.60	0.017935705
ENSMUSG00000022652	MORC1	16.81	5.97	0.36	0.021711344
ENSMUSG00000005470	ASF1B	50.15	29.63	0.59	0.023886394
ENSMUSG00000022432	SMC1B	25.64	11.30	0.44	0.025196064
ENSMUSG00000030041	D6Mm5e	29.26	14.78	0.51	0.028344916
ENSMUSG00000020766	Galk1	53.81	32.27	0.60	0.029888826
ENSMUSG00000009670	Tex11	33.19	17.97	0.54	0.030252176
ENSMUSG00000015365	Mov10l1	64.02	41.85	0.65	0.033078693
ENSMUSG00000040152	Thbs1	15.75	5.29	0.34	0.033452824
ENSMUSG00000070348	CCND1	25.78	12.78	0.50	0.041242165
ENSMUSG00000023443	ESX1	11.19	3.35	0.30	0.044683493
ENSMUSG00000093599	Mir5620	5.38	0.92	0.17	0.049303386
ENSMUSG00000085666	Gm9855	13.89	0.00	NA	1.22E-05
ENSMUSG00000059040	eno1	6.29	0.00	NA	0.001980656
ENSMUSG00000098453	Mir6997	3.54	0.00	NA	0.019378059
ENSMUSG00000093404	Gm25190	2.11	0.00	NA	0.042875493
ENSMUSG00000080384	Gm22105	2.16	0.00	NA	0.042875493
ENSMUSG00000027073	PRG2	2.22	0.00	NA	0.042875493

Up-regulated in *Bcas2$^{F/-}$;V-cre* sample

GeneNames		RPKM-control	RPKM-*Bcas2$^{F/-}$;V-cre*	FC-normalized	p-value
ENSMUSG00000085354	Gm2044	160.64	260.18	1.62	3.55E-07
ENSMUSG00000003555	CYP17A1	94.79	152.09	1.60	0.000116227
ENSMUSG00000065126	Snord104	64.55	101.98	1.58	0.002687811
ENSMUSG00000029304	SPP1	54.31	84.33	1.55	0.007791217
ENSMUSG00000067608	Pcna-ps2	0.08	6.79	85.01	0.02284126
ENSMUSG00000034674	TDG	40.75	62.35	1.53	0.023503576
ENSMUSG00000088929	Gm24299	28.99	47.36	1.63	0.023688103
ENSMUSG00000077565	Gm23201	5.64	15.69	2.78	0.026625954
ENSMUSG00000093124	Gm24366	0.58	5.41	9.27	0.043878831
ENSMUSG00000064542	Gm22980	1.43	7.39	5.15	0.046219061
ENSMUSG00000064390	Rnu73b	0.00	4.37	NA	0.008015242
ENSMUSG00000098363	Mir6993	0.00	3.18	NA	0.017715641
ENSMUSG00000089265	Gm22888	0.00	3.36	NA	0.017715641
ENSMUSG00000098479	Gm27263	0.00	2.04	NA	0.039996352
ENSMUSG00000065439	Mir140	0.00	2.22	NA	0.039996352
ENSMUSG00000093119	Gm25640	0.00	2.07	NA	0.039996352
ENSMUSG00000099107	Gm27898	0.00	2.59	NA	0.039996352
ENSMUSG00000092953	Gm26217	0.00	2.46	NA	0.039996352
ENSMUSG00000094650	Gm25361	0.00	2.04	NA	0.039996352
ENSMUSG00000064770	n-R5s29	0.00	2.61	NA	0.039996352

FC (Fold Change): RPKM-*Bcas2$^{F/-}$;V-Cre* /RPKM-Control.

FC>1.5 as the threshold for significantly differential expression

P-value: Corrected P-value <0.05 as the threshold for significantly differential expression

Supplementary Table 4. A list of changed alternative splicing events

AccID	Location	Exon_ Number	Control_Skip ::Others	*Bcas2F/-;V-Cre* _Skip::Others	CExp	FVExp	Adjusted_ PValue	SplicingType
2610005L07Rik	8:20401377-20401496	9	107::490	421::374	49::27	13::56	1.55E-08	cassette
Gm21811	8:20401377-20401496	8	10::490	327::374	49::24	13::45	1.84E-07	cassette
2610005L07Rik	8:20278090-20278170	17	47::132	130::102	57::27	21::56	2.33E-07	altend
2610005L07Rik	8:20271922-20272002	21	15::0	22::98	5::27	76::56	1.42E-05	altend
Casp2	6:42276870-42276930	9	98::6	79::43	2::107	17::98	1.72E-04	cassette
Dym	18:75126664-75126744	15	95::0	119::20	1::83	19::67	2.29E-04	altstart
2610005L07Rik	8:20405121-20405195	8	255::0	188::302	71::105	14::71	3.02E-04	alt5
Magea5	x:155055395-155055483	5	0::82	6::10	35::19	7::16	0.001853	cassette
Smpd4	16:17620586-17620630	3	78::1	76::25	1::77	10::73	0.001944	cassette
Ints10	8:68824190-68824267	17	173::111	141::14	59::82	17::58	0.002032	cassette
Fam135a	1:24042949-24043024	14	47::1	52::22	1::74	12::78	0.00221	cassette
Adck5	15:76604626-76604671	51	108::0	82::12	4::109	13::87	0.002313	cassette
E2f5	3:14581136-14581206	2	62::16	41::50	7::100	22::95	0.002344	cassette
Wbp1l	19:46606712-46606759	2	50::103	37::205	36::130	83::140	0.002404	cassette
Gm10256	y:2832910-2833060	3	360::134	235::144	74::13	72::1	0.002712	cassette
Dazl	17:50284996-50285046	8	103::1622	260::403	574::771	202::560	0.002863	cassette
Gm17606	14:54649522-54649532	14	15::113	2::127	47::70	132::85	0.00306	alt3
Acin1	14:54649522-54649532	20	15::113	2::127	47::70	132::85	0.00306	alt5
Gls	1:52200618-52200651	10	339::20	208::36	52::73	79::58	0.003205	cassette_multi
Ppm1b	17:85023032-85023079	9	20::79	18::18	35::164	11::170	0.003226	cassette
Gm21811	8:20405121-20405195	10	255::0	188::302	71::86	14::43	0.003357	alt3
Srbd1	17:85997506-85997547	20	45::6	40::27	3::52	11::40	0.003815	cassette
Cpsf1	15:76604626-76604671	2	108::0	82::12	4::116	13::90	0.003858	cassette
Gm27029	11:101416849-101416925	10	92::6	88::22	3::100	15::83	0.004002	cassette
1810058I24Rik	6:35252956-35253045	2	505::52	516::119	46::165	94::159	0.004259	cassette
Aarsd1	11:101416849-101416925	3	92::6	88::22	3::113	15::92	0.004417	cassette
Gnaz	10:74972197-74972243	2	24::0	16::18	1::20	8::16	0.00454	cassette
Trrap	5:144810151-144810204	34	109::6	124::41	6::116	19::120	0.005066	cassette_multi
Smpd4	16:17620712-17620762	4	76::9	76::34	4::77	14::73	0.005068	cassette_multi
Ska1	18:74207530-74207594	1	9::45	20::20	7::30	16::16	0.005269	alt5
Lnp	2:74534615-74534668	13	23::21	23::1	9::31	1::32	0.005345	cassette
Tbc1d1	5:64284690-64284809	15	41::1	37::18	2::42	12::39	0.005408	cassette_multi
Hk1	10:62291975-62292043	19	67::4	60::29	2::70	12::76	0.005424	cassette
2310045N01Rik	8:70159386-70159454	4	36::2	22::19	2::32	9::24	0.00583	cassette
Ddi2	4:141689270-141689331	11	32::93	11::198	38::42	78::45	0.006111	cassette
Ptbp2	3:119760651-119760731	4	84::9	95::38	11::128	27::121	0.00617	altend
Rdh13	7:4443737-4443771	4	43::1	35::29	2::27	10::25	0.006253	cassette
Rgs3	4:62626492-62626531	16	5::7	31::5	3::28	15::30	0.006305	mutually_exclusive
Rsc1a1	4:141689270-141689331	11	32::93	11::198	38::73	78::81	0.006318	cassette
Esrp1	4:11338722-11338831	17	60::43	32::65	17::70	32::58	0.00635	cassette

Mef2b	8:70159386-70159454	3	36::2	21::19	2::29	9::22	0.006398	cassette
Eif2b3	4:117070942-117071022	13	240::11	176::24	11::155	23::106	0.006876	altend
Rgs3	4:62626492-62626531	6	5::7	31::5	3::47	15::55	0.006926	mutually_exclusive
Copz2	11:96852111-96852154	3	110::75	116::25	25::138	8::119	0.006961	cassette
Polr3h	15:81917474-81917594	5	167::6	149::18	3::80	19::68	0.007533	cassette
Ascc1	10:60002418-60002467	5	197::60	190::102	35::120	58::100	0.007541	cassette_multi
Rplp0	5:115561099-115561245	4	5510::1563	2694::153	2146::3710	723::2594	0.008056	unknown
6720401G13Rik	x:50606076-50606162	7	12::33	31::16	21::57	10::68	0.008311	unknown
Cinp	12:110878988-110879039	4	111::12	94::29	25::90	43::77	0.008448	cassette
Atf2	2:73870623-73870685	4	139::34	134::92	13::95	33::106	0.008786	cassette
Smim14	5:65485054-65485127	3	269::32	280::8	14::55	5::62	0.00899	cassette
Zfp518a	19:40894705-40894790	2	19::5	15::23	3::107	11::93	0.009223	altstart
Il15ra	2:11727763-11727833	7	9::1	18::27	1::25	9::19	0.009236	cassette
Trim34b	7:104353194-104353274	10	39::17	29::37	12::38	38::48	0.009293	altend
E130114P18Rik	4:97584548-97584607	5	0::58	11::29	37::30	26::44	0.009611	altstart
Gtf2a1	12:91585375-91585478	4	103::5	78::18	2::83	10::76	0.009629	cassette
Ccdc152	15:3293853-3293921	4	7::35	0::89	16::19	42::21	0.009846	cassette_multi
Ap4m1	5:138172437-138172787	3	107::21	59::48	9::88	21::71	0.010073	retain_intron
Mitd1	1:37879997-37880088	6	101::2	62::14	3::41	12::39	0.010239	cassette
Fam105a	15:27680404-27680434	2	33::0	43::14	3::27	12::25	0.010256	cassette
Rfc4	16:23114107-23114187	11	151::14	76::54	21::131	35::91	0.010351	retain_intron
Tsc22d3	x:140542511-140542846	2	28::28	83::23	5::21	15::13	0.010525	alt5
Bnip1	17:26783244-26783332	3	117::1	150::18	9::75	23::77	0.010653	cassette
Armc9	1:86218848-86218898	21	103::0	55::11	1::24	6::19	0.010841	cassette
Hspb11	4:107254918-107255020	4	133::4	116::14	2::74	15::73	0.01152	cassette
Tial1	7:128458597-128458656	3	173::55	131::139	27::80	57::97	0.01162	cassette
Zcchc2	1:106006015-106006086	4	7::15	1::47	6::21	19::22	0.012305	cassette
Mecr	4:131861177-131861232	6	103::17	95::43	11::68	22::63	0.012346	cassette
Socs5	17:87120748-87120808	2	13::1	10::14	3::42	12::43	0.012392	cassette_multi
Myh14	7:44657417-44657440	6	5::3	1::22	1::10	8::10	0.012469	cassette
Chd1l	3:97580979-97581003	15	5::96	19::67	9::111	19::86	0.012609	alt5
Gm527	12:64922682-64922768	5	63::11	33::22	8::53	17::43	0.01272	cassette
Golga4	9:118579542-118579606	26	104::58	104::17	38::63	21::64	0.012838	cassette
Palm	10:79816792-79816923	8	105::77	70::97	29::94	47::77	0.012846	cassette
Myo9b	8:71336876-71336965	15	9::83	14::37	39::35	16::33	0.013066	cassette
Gm20431	2:167629069-167629288	8	319::20	308::78	10::124	27::123	0.013115	cassette
Prrg1	x:78506914-78506979	4	29::0	32::17	1::42	8::46	0.013238	cassette
Ret	6:118162391-118162471	19	67::16	29::20	15::29	31::24	0.013292	altend
Dtx2	5:136026470-136026607	9	71::53	31::76	22::99	36::81	0.013452	cassette_multi
Ccdc36	9:108412493-108412580	6	0::76	6::16	32::32	8::21	0.013531	cassette
Zfp710	7:80088535-80088632	6	47::7	27::16	7::50	19::47	0.013705	cassette
Nup107	10:117768254-117768323	18	171::6	162::19	2::97	10::87	0.013817	cassette
Mms22l	4:24521098-24521144	11	20::1	23::17	1::35	7::34	0.013955	cassette

Mllt4	17:13848355-13848375	17	35::8	25::43	5::77	12::71	0.01403	cassette
Ankrd12	17:65982072-65982149	13	74::4	55::16	6::60	14::50	0.014268	cassette
Polr3g	13:81693846-81693890	4	247::19	199::39	20::145	36::132	0.014381	altend
Atm	9:53440747-53440873	3	102::4	83::24	4::41	12::34	0.014517	cassette
Anapc16	10:60002418-60002467	8	75::60	56::102	35::129	58::123	0.014598	unknown
Dnm3	1:162273604-162273649	15	8::33	6::3	8::7	1::8	0.014675	cassette
Ythdf3	3:16203187-16203219	5	51::72	51::135	38::145	82::164	0.014768	cassette
Uros	7:133708924-133709009	2	6::34	32::30	8::16	22::12	0.015587	alt5
Magea2	x:155055395-155055483	6	0::82	6::10	35::7	7::5	0.015828	cassette
Pradc1	6:85447688-85447807	5	22::7	14::15	9::56	21::34	0.01586	alt3
Gtf2a1	12:91573299-91573352	6	73::4	60::17	9::83	18::76	0.016125	cassette
1600020E01Rik	6:86541472-86541546	5	77::0	68::10	1::43	6::35	0.016279	cassette
Plekhg2	7:28364509-28364585	18	129::9	120::37	11::76	28::81	0.01635	retain_intron
Cbx5	15:103215022-103215125	3	111::54	103::22	22::124	10::142	0.016912	cassette
Ampd2	3:108085247-108085399	3	28::5	32::21	4::79	15::67	0.017115	altstart
Hspbap1	16:35792986-35793066	3	80::0	47::7	1::25	7::23	0.017235	altstart
Gm10256	y:2865206-2865318	6	7::0	1::81	91::13	54::1	0.017325	mutually_exclusive
Gm10256	y:2834115-2834153	5	0::110	81::69	41::13	19::1	0.017609	mutually_exclusive
Sh2b1	7:126469488-126469570	7	93::3	107::15	8::113	27::99	0.017624	cassette
Hdac10	15:89126630-89126641	8	7::30	16::14	4::26	13::25	0.018004	alt5
9330136K24Rik	17:21535823-21535878	13	24::0	37::20	3::5	12::4	0.018092	unknown
Ppp1r15a	7:45523461-45523739	1	12::61	26::51	6::57	16::45	0.018206	alt5
Lig3	11:82800499-82800653	20	77::23	101::1	11::80	2::71	0.018299	altend
Wdr83	8:85076132-85076217	9	77::17	73::54	13::72	32::79	0.018389	retain_intron
Commd8	5:72164755-72164854	4	152::69	188::44	33::66	21::78	0.019167	cassette
Acsl3	1:78664326-78664406	3	69::8	73::26	12::165	23::125	0.019273	altend
Adat1	8:111990396-111990412	3	0::20	10::4	21::26	7::26	0.019293	alt3
2700099C18Rik	17:94761498-94761604	7	20::101	46::78	63::73	46::92	0.019297	cassette_multi
Tsc22d2	3:58428228-58428299	2	76::29	53::47	8::37	18::34	0.019324	cassette
Gm21811	8:20399303-20399468	6	44::217	349::164	18::24	14::45	0.019406	cassette
R3hdm2	10:127465131-127465182	19	108::26	88::3	18::98	6::95	0.01955	altend
Cdk2	10:128731324-128731412	11	0::52	6::22	26::86	11::76	0.019682	cassette
Brf1	12:112983733-112983849	6	174::12	160::35	10::120	24::125	0.019743	cassette
Ctns	11:73197099-73197252	2	41::35	57::9	18::35	8::41	0.020046	cassette
Ell	8:70539412-70539492	2	2::7	7::0	14::69	4::61	0.020218	altstart
Bicc1	10:70953102-70953182	11	39::20	36::4	20::50	8::50	0.020229	altend
Ash1l	3:89030240-89030352	8	55::15	77::0	10::52	3::57	0.020245	cassette
Slmo1	18:67477950-67478061	6	16::12	13::44	11::22	28::23	0.020569	cassette
Adat1	8:111991925-111992005	2	20::15	14::1	21::16	5::17	0.020768	altend
Kif21a	15:90951246-90951374	30	52::52	80::22	24::75	13::84	0.020893	cassette
2310045N01Rik	8:70158814-70158894	3	29::7	13::17	8::32	15::24	0.021344	altstart
Pbrm1	14:31056236-31056280	19	67::39	48::78	15::96	27::100	0.021399	cassette
Epb4.1	4:131962924-131962986	18	22::70	31::43	32::99	15::94	0.021418	cassette_multi
Gclm	3:122261668-122261796	6	170::11	117::44	17::28	34::26	0.021635	cassette

Gm21811	8:20277872-20277945	4	0::5	16::2	31::24	101::45	0.022089	mutually_exclusive
Hmga1	17:27556952-27557066	6	613::79	431::23	58::188	19::126	0.022758	cassette
Mef2b	8:70158814-70158894	2	29::7	12::17	8::29	15::22	0.022869	altstart
Parp6	9:59641741-59641810	20	57::8	61::24	8::50	22::48	0.023319	cassette
Fam160a2	7:105391577-105391667	3	38::6	35::31	7::21	19::22	0.023427	cassette_multi
Mapk8ip3	17:24901529-24901605	24	37::1	33::18	2::51	11::52	0.023449	retain_intron
B3gntl1	11:121651609-121651673	5	12::27	7::71	12::41	26::45	0.023509	cassette_multi
Dgka	10:128731324-128731412	10	0::52	6::22	26::29	11::27	0.023548	cassette
Recql	6:142386862-142386942	2	50::11	50::0	8::62	1::55	0.023641	altstart
Ptgr2	12:84296228-84296356	4	136::6	148::25	4::78	13::81	0.023797	cassette
Papd4	13:93148366-93148473	16	173::50	185::17	42::128	24::138	0.024069	cassette
Col4a3bp	13:96616994-96617071	12	139::210	130::125	74::92	46::94	0.024459	cassette
Sh3d21	4:126152118-126152206	12	27::9	10::16	4::21	11::14	0.024487	retain_intron
Dnajc5	2:181521235-181521287	3	113::51	94::100	20::122	39::134	0.024789	cassette
Sepp1	15:3293853-3293921	10	7::35	0::89	16::378	42::360	0.024928	cassette_multi
Dcun1d2	8:13282534-13282580	2	12::15	20::0	7::52	2::50	0.025468	cassette
Hmga1	17:27556711-27556749	5	434::101	328::25	94::223	35::159	0.02559	alt5
Gclm	3:122261668-122261868	6	11::166	36::114	17::168	26::110	0.026207	alt3
Utp14a	x:48273899-48273976	12	31::125	16::178	63::69	89::62	0.026306	cassette_multi
Kdm5d	y:927603-927710	15	9::4	6::17	12::24	6::40	0.026364	alt3
Ube3a	7:59240967-59241033	2	0::8	15::1	9::86	20::94	0.026486	mutually_exclusive
Gm26782	14:50871751-50871826	2	32::20	48::10	15::20	7::26	0.026619	cassette
Gba	3:89206760-89206812	9	66::4	64::18	9::106	17::102	0.026691	cassette
BC029214	2:25459859-25459915	6	72::14	88::5	26::46	13::55	0.027011	alt3
Lipo1	19:33582093-33582251	9	18::28	0::22	28::18	30::41	0.027277	alt3
Ppp2r5c	12:110574745-110574861	18	15::0	7::6	26::114	11::102	0.027319	mutually_exclusive
Plce1	19:38721920-38721968	13	51::1	35::7	6::48	16::43	0.027376	alt5
Fbxl2	9:113969922-113969977	7	133::5	106::25	21::7	37::5	0.027838	cassette
Csnk1g3	18:53936853-53936948	11	77::38	96::18	18::86	9::92	0.027871	cassette
Cpsf3l	4:155888109-155888184	14	192::4	144::29	17::113	27::82	0.027879	retain_intron
4930533K18Rik	10:70953102-70953182	20	39::20	36::4	20::35	8::33	0.028095	altstart
Nsl1	1:191083637-191083691	9	12::2	15::29	3::20	17::25	0.028108	retain_intron
Kdelc1	1:44101900-44101957	2	221::305	211::175	149::331	82::287	0.028125	cassette_multi
Lrrc8b	5:105415775-105415912	2	0::26	5::17	21::27	9::32	0.028456	altstart
Cntln	4:84893380-84893460	4	56::3	44::12	3::45	11::47	0.028495	altend
Plekha1	7:130909628-130909735	13	90::11	42::22	39::90	55::74	0.02866	alt5
Disp1	1:183173496-183173623	4	11::8	0::10	55::28	29::28	0.028798	mutually_exclusive
Cmbl	15:31572188-31572253	2	20::0	14::7	1::31	7::34	0.028975	altstart
Nup214	2:31992910-31992990	13	31::1	12::8	2::39	7::35	0.029025	altend
Hecw2	1:53933217-53933288	8	6::5	0::15	7::15	14::9	0.029073	alt3
Tank	2:61629285-61629372	7	73::0	82::11	1::59	7::66	0.029265	cassette
AA465934	11:83291833-83291967	1	270::21	148::46	21::111	27::71	0.029381	retain_intron
Gm15559	5:110776554-110776607	4	61::24	74::9	15::34	6::31	0.029577	cassette
1810058I24Rik	6:35252782-35253045	1	507::50	525::110	39::139	73::139	0.029669	alt5

Ube2v1	2:167629069-167629288	2	310::21	302::79	10::171	27::168	0.029991	cassette
Evi5	5:107810290-107810322	19	105::31	106::62	14::119	26::126	0.030243	cassette_multi
Ppp1r15a	7:45523461-45523739	1	12::61	26::51	6::59	16::52	0.030776	alt5
Dusp19	2:80624208-80624326	4	52::5	33::18	48::32	42::49	0.030919	alt3
Nup35	2:80624208-80624326	3	52::5	33::18	48::32	42::49	0.030919	alt3
Foxp1	6:99399605-99399717	5	15::1	5::16	1::36	5::28	0.031104	cassette
Chordc1	9:18308222-18308284	9	162::65	203::34	31::149	19::163	0.031121	cassette
Scml2	x:161228677-161228760	27	144::478	121::279	74::223	31::187	0.031283	cassette_multi
Kif21a	15:90993701-90993742	6	32::6	50::0	31::50	47::42	0.031442	alt5
Cnot3	7:3658745-3658824	18	171::8	199::23	5::109	20::116	0.031599	altstart
Selo	15:89092849-89092907	2	14::20	2::39	23::31	39::26	0.031735	alt5
Svil	18:5104341-5104392	37	78::11	59::0	15::47	7::48	0.031784	cassette
Kmt2b	7:30570468-30570554	33	100::17	106::50	13::94	32::105	0.03181	retain_intron
Ptprd	4:76095520-76095546	19	27::12	26::34	3::8	12::9	0.032238	cassette
9130011E15Rik	19:45835573-45835653	25	33::10	39::1	11::44	3::39	0.032473	altstart
Cfp	x:20927181-20927281	8	1::38	15::26	6::40	12::28	0.032493	alt5
Ano1	7:144648575-144648640	8	17::0	27::15	1::14	6::13	0.032736	cassette
2610005L07Rik	8:20272139-20272219	20	15::159	121::182	61::27	78::56	0.032739	altend
Brd3	2:27453400-27453450	15	144::112	147::58	49::176	28::166	0.03286	cassette
Dis3	14:99099119-99099186	2	38::9	37::0	6::18	1::19	0.033235	cassette
Lgals4	7:28840610-28840864	7	11::25	30::7	17::23	6::25	0.033315	retain_intron
Cyth4	15:78604860-78604940	6	10::5	25::0	7::13	1::12	0.033407	altend
Cit	5:115933645-115933655	17	3::8	6::0	7::12	1::10	0.033432	altstart
Spin2c	x:153832329-153832435	1	12::3	3::15	3::14	11::11	0.033507	retain_intron
Ubp1	9:113969922-113969977	16	133::5	106::25	21::135	37::142	0.033595	cassette
Lmbr1	5:29274218-29274298	12	106::4	70::10	5::33	11::23	0.033734	altend
Gm9183	17:47680190-47680238	15	67::0	52::10	6::77	11::71	0.033978	cassette
Mms19	19:41945388-41945459	29	203::33	150::71	27::148	45::127	0.033987	retain_intron
St3gal4	9:35126212-35126307	8	19::201	36::175	24::204	51::200	0.034296	alt5
Kif13b	14:64800345-64800446	38	11::25	4::43	6::27	17::29	0.034344	cassette
2210015D19Rik	11:5765509-5765571	2	16::64	4::112	25::22	46::24	0.034524	cassette_multi
Kmt2a	9:44843198-44843244	8	8::9	12::0	5::29	1::28	0.03501	cassette
Kctd9	14:67734141-67734290	10	59::85	72::45	58::101	34::102	0.03538	cassette
Abcc5	16:20395541-20395620	12	27::1	37::16	4::64	12::71	0.035405	cassette
Macf1	4:123469723-123469872	39	16::5	7::15	2::53	9::56	0.035462	cassette
Ogdh	11:6316765-6316855	6	45::59	19::59	29::127	14::127	0.035597	mutually_exclusive
Usp33	3:152374891-152374915	15	28::73	58::36	54::119	74::104	0.036334	alt3
BC029214	2:25459859-25459915	6	72::14	88::5	26::81	13::85	0.036568	alt3
Casc3	11:98801966-98802027	4	4::99	13::92	15::147	33::139	0.03658	mutually_exclusive
Klhl15	x:94253940-94254067	5	38::26	13::33	19::51	31::46	0.036668	altend
Hps1	19:42779738-42779914	1	4::16	12::7	4::8	9::3	0.036682	alt5
Zfp651	9:121765945-121765997	4	67::9	66::0	10::43	5::49	0.036733	cassette
Slc8b1	5:120530316-120530396	15	2::52	5::17	24::22	10::20	0.036985	cassette_multi
Pus1	5:110776554-110776607	4	59::24	69::9	15::65	6::54	0.037187	cassette

Chid1	7:141501540-141501595	15	114::11	93::0	6::60	1::50	0.037304	cassette
Grlf1	7:16598878-16598934	3	21::5	22::19	2::69	9::69	0.037497	cassette
Kif1a	1:93063632-93063712	17	28::15	46::6	13::33	6::36	0.037611	altstart
Hsf1	15:76499132-76499215	10	64::46	80::16	26::89	15::90	0.037641	cassette
Gm5918	9:72982560-72982626	2	87::4	64::20	5::60	10::54	0.037647	cassette
Ahdc1	4:133060676-133060781	7	18::18	7::26	14::20	7::29	0.037796	alt3
Nsfl1c	2:151502455-151502460	6	331::701	224::784	103::414	127::357	0.03784	cassette
Nsun5	5:135375485-135375555	8	93::40	62::70	27::110	42::92	0.038574	retain_intron
Ptprk	10:28562107-28562142	16	34::1	41::13	1::28	6::33	0.038706	cassette
Chd1l	3:97574047-97574112	16	98::3	77::12	8::119	13::95	0.038867	cassette
Dleu2	14:61632437-61632483	6	0::23	3::5	10::6	5::8	0.039114	cassette
Emg1	6:124705088-124705158	4	84::85	47::169	54::178	94::172	0.039189	retain_intron
Znrd1	17:36958296-36958378	3	203::72	191::23	59::60	28::52	0.039576	retain_intron
Mgat1	11:49251550-49251674	4	28::156	9::227	92::73	125::62	0.039816	cassette
Mageb18	x:92240410-92240469	5	9::8	20::0	4::13	1::16	0.039895	cassette
Skiv2l	17:34845100-34845168	14	34::15	24::42	13::53	24::46	0.040017	retain_intron
Pde8b	13:95085173-95085261	12	18::57	41::38	19::36	11::40	0.040019	cassette_multi
Gstm6	3:108042327-108042411	6	61::0	50::327	43::41	39::58	0.040023	alt5
Gstm3	3:108042327-108042411	6	61::0	50::327	43::41	39::58	0.040023	alt5
Gm12494	3:108042327-108042411	6	61::0	50::327	43::41	39::58	0.040023	alt5
Mtmr2	9:13760418-13760488	2	57::39	44::75	17::123	33::135	0.040306	cassette
Tcf4	18:69678117-69678129	26	88::72	74::31	80::114	44::106	0.040685	alt5
Sipa1l3	7:29331948-29332043	21	44::16	31::31	7::43	15::41	0.040911	cassette
Pik3cd	4:149675912-149676044	5	5::14	9::3	12::22	4::23	0.041425	altstart
Bfar	16:13690258-13690329	5	60::8	82::1	15::81	7::78	0.041444	cassette
Zkscan2	7:123482641-123482684	7	14::1	3::5	1::11	4::9	0.041477	cassette
Vps33b	7:80277644-80277697	6	50::8	42::0	7::33	1::30	0.041516	cassette
Aff3	1:38318524-38318604	12	30::0	24::6	1::39	6::37	0.041553	altend
Trim33	3:103353567-103353617	19	40::227	55::164	83::66	70::76	0.04156	cassette
Zfp868	8:69619314-69619671	2	128::60	108::80	74::101	49::122	0.041645	unknown
Fam149b	14:20367846-20368563	8	76::38	53::66	32::67	51::60	0.041896	altend
Jmjd1c	10:67216983-67217107	6	14::17	8::42	14::80	26::82	0.042151	cassette
Mthfs	9:89211401-89211481	4	45::73	59::52	37::24	17::22	0.042376	altstart
Zmynd8	2:165807519-165807675	25	16::17	6::24	14::29	5::33	0.042527	alt5
Gnb1l	16:18540435-18540575	6	9::105	17::59	47::13	20::12	0.042563	cassette_multi
Snx13	12:35119733-35119766	18	33::109	19::134	34::70	21::79	0.04263	alt3
Myo9b	8:71348901-71348935	23	27::80	47::33	51::83	29::85	0.042701	retain_intron
2610020H08Rik	7:119794502-119794593	1	23::0	50::9	1::32	11::36	0.042873	alt5
Erc1	6:119694284-119694364	19	45::0	29::5	5::31	11::26	0.042939	altend
Exoc1	5:76557819-76557863	12	151::143	118::73	59::144	37::144	0.043101	cassette
Csnk1g3	18:53948645-53948668	13	43::221	64::180	68::86	52::92	0.043156	cassette
Arrb2	11:70435516-70435542	3	22::209	37::130	50::212	54::150	0.043416	alt3
Bcl2l11	2:128127575-128127812	2	11::16	23::11	14::23	3::21	0.043479	altstart
Gpatch11	17:78838250-78838383	4	163::7	135::17	5::50	12::44	0.04392	cassette

Synj1	16:90947015-90947053	29	10::13	11::1	6::15	2::16	0.044223	cassette_multi
Zpbp	11:11429063-11429126	3	6::19	5::1	9::6	1::4	0.044245	cassette
2610005L07Rik	8:20274162-20274242	18	32::233	121::374	43::27	54::56	0.044309	altend
Brf1	12:112987137-112987192	4	120::2	77::21	33::120	51::125	0.044591	cassette
Adarb1	10:77312348-77312378	10	5::23	10::3	9::25	9::9	0.044617	alt5
1700007J10Rik	11:59737412-59737582	21	1::8	6::3	14::145	30::138	0.044668	mutually_exclusive
Rapgef4	2:72172786-72172866	9	1::5	7::0	6::9	2::12	0.044758	altstart
Rffl	11:82811028-82811111	8	31::86	41::41	42::67	21::54	0.04477	cassette
Ubl7	9:57911007-57911059	1	1::96	10::75	51::6	40::10	0.044949	alt5
Abi1	2:22963214-22963228	5	115::263	74::326	73::213	93::196	0.045048	cassette
Cmc1	9:118068674-118068828	5	260::14	221::29	17::76	27::67	0.045386	cassette
Kifc3	8:95100647-95100673	23	7::51	0::67	18::29	25::25	0.045492	cassette
Ehmt2	17:34905610-34905711	10	181::931	372::610	361::404	232::388	0.045702	cassette
Cpsf3l	4:155888326-155888396	15	109::12	60::32	14::108	21::79	0.045749	retain_intron
Tbc1d1	5:64283967-64284125	14	41::2	36::17	3::43	9::40	0.046223	cassette_multi
Gm20695	16:3907999-3908134	9	25::0	19::5	1::47	7::45	0.046378	cassette
Tulp2	7:45523461-45523632	29	60::12	51::26	9::179	24::193	0.046397	altend
Trappc13	13:104152165-104152182	9	78::98	91::53	25::92	15::83	0.047398	cassette
Rsbn1l	5:20908212-20908244	4	2::51	14::44	48::36	51::61	0.047487	alt3
Rcbtb1	14:59209743-59209810	4	48::3	39::17	12::98	21::103	0.047494	cassette
E2f3	13:29984128-29984219	2	15::3	3::8	2::30	7::30	0.047706	altstart
Gm20517	17:47680190-47680238	12	67::0	52::10	6::38	11::38	0.04775	cassette
Gm2694	8:87513809-87513896	3	76::39	65::15	18::39	6::31	0.047983	cassette
Sh3bgr	16:96205719-96205939	2	96::75	91::123	23::129	46::133	0.048021	altstart
Cblb	16:52186349-52186480	18	15::16	37::3	12::44	5::45	0.048189	cassette
Dpysl4	7:139088365-139088398	2	127::5	142::29	21::112	43::132	0.048259	altstart
Myzap	9:71586172-71586216	2	83::11	95::28	3::42	9::36	0.048472	cassette
Pibf1	14:99099119-99099186	2	38::9	37::0	6::39	1::32	0.048497	cassette
Mirg	12:109743357-109743418	15	0::11	2::0	6::7	1::6	0.048504	cassette
Svil	18:5057275-5057439	15	33::1	28::8	1::48	7::49	0.048558	cassette
Zfp382	7:30130056-30130143	3	8::0	8::17	1::8	7::10	0.048584	cassette
Nudt6	3:37415651-37415692	4	62::3	34::11	2::26	6::29	0.048863	cassette
Supt16	14:52212719-52212743	25	52::32	79::24	40::97	27::120	0.048926	alt3
Gm20535	12:21256475-21256609	31	3::11	6::3	10::86	2::79	0.048955	cassette
Tmem68	4:3562020-3562146	9	22::13	30::1	7::40	2::42	0.048976	cassette
Ggta1	2:35414219-35414284	9	4::29	8::13	14::18	7::22	0.049202	cassette
Zfp652	11:95825195-95825293	10	43::12	31::0	7::27	1::22	0.04931	cassette
Cdc14b	13:64201332-64201448	16	32::25	17::30	58::66	38::77	0.049341	mutually_exclusive
Rnf111	9:70429655-70429679	15	81::20	57::32	33::83	42::65	0.049559	alt3
Ndufaf5	2:140199365-140199460	10	97::27	84::52	17::59	26::54	0.049738	cassette
Fam76a	4:132909626-132909754	8	128::2	128::20	7::120	16::123	0.04993	cassette
Map2k4	11:65781353-65781433	2	95::12	81::34	25::148	40::151	0.049976	altend

Supplementary Table 5. Primer sequence used for real-time RT-PCR assay

Gene	Forward (5'→3')	Reverse (5'→3')
Bcas2	TCGCTGCTCGACAACCGATTGAA	AGCTGCATGTTCTTTCGCTGCCA
Gapdh	CCCCAATGTGTCCGTCGTG	TGCCTGCTTCACCACCTTCT
Oct4	GAAGCAGAAGAGGATCACCTTG	TTCTTAAGGCTGAGCTGCAAG
Sox2	GGCAGCTACAGCATGATGCAGGAGC	CTGGTCATGGAGTTGTACTGCAGG
c-Kit	TCATCGAGTGTGATGGGAAA	GGTGACTTGTTTCAGGCACA
Hprt	GCCAGTAAAATTAGCAGGTGTTCT	ATAGGCTCATAGTGCAAATCAAAAG
Nanos2	GGAATAAGAGGAAGGTACAGGAACTA	TATATTGGATGGGTAGAAGAGAGAGAA
Stra8	CTGTTGCCGGACCTCATGG	TCACTTCATGTGCAGAGATGATG
Dmc1	AGCCACCTTCGCAATAACC	CCACTTCTCCAGCCCCTAAT
Rec8	CTACCTAGCTTGCTTCTTCCCA	GCCTCTAAAAGGTGTCGAATCTG
Mvh	CTAGGAAGACCAAATAGTGAATCTGAC	TCCAGAACCTGTTACTACTTCTTCATT
Sycp1	AAGTTTGATTCTAAAACAACTCCTTCA	ACTCTTTTTAGTTGGTGTCTTCACTGT
Sycp3	AGAAATGTATACCAAAGCTTCTTTCAA	TTAGATAGTTTTTCTCCTTGTTCCTCA
Sohlh2	TCTCAGCCACATCACAGAGG	GGGGACGCGAGTCTTATACA
Sohlh1	GGGCCAATGAGGATTACAGA	CACAGGAGCTGTGCAGAGAG
Tuba3a-premRNA	CCACTACACCATCGGCAAAG	CCAGACAGCCACCATCTAAAGC
Tuba3a-mRNA	GGATGTGGTTCCCAAAGATGT	CACAGTGGGAGGCTGGTAGTT
Tuba3b-premRNA	AGCCAACAATTATGCCAGAGG	CCTTGACCGGGACAACCAT
Tuba3b-mRNA	CACAGGCCGATCTGTGCA	CCACTGAAAGCCGCTCCAT
Tubb4b-premRNA	GGACTCGGTGCGTTCAGG	GCTTTCGGCCAAGGGATT
Tubb4b-mRNA	AGCTGTTCCGCAGTCGCC	GGTCGCTATCTCCGTGGTAAG
Dazl	GGATGAAACCGAAATCAGGA	ATAGCCCTTCGACACACCAG
Dazl-FL	TCCACCACAGTTCCAGAGTG	AACATAACTCCTCTGCTCTCCA
Dazl-Δ 8	TCCACCACAGTTCCAGAGTG	CAGTTGTATAAGCCTGGTAG
Dazl+7	TGTGCAGGCCCCGTGTAT	GGAACCCTGGGGAAGAAGA
Hmga1	GAGCCCAGTGAAGTGCCAAC	CCTTGGTTTCCTCCCTGGAG
Hmga1-E1	CCCCAGCTCGGATGTGAC	GCAAATGCGGATCTGAAACC
Ehmt2	CATCAATGCCGTAGATAAGCAA	GTGGAGCCATCCTCTTCCTT
Ehmt2-Δ10-F	AGCCAAGAGGGGTCTCCAA	CTCGCTGATGCGGTCAATC
Ehmt2-FL-F	AGCCAAGAGGAGTGAATGGTGT	CCAGTGAAGACGTGTCATTGGA

Supplementary Table 6. Primer sequence used for RT-PCR assay

Gene	Gene Forward (5'→3')	Reverse (5'→3')
Dazl	TCCTCCTTATCCAAGTTCACCA	TCAGCTCCTGGATCAACTTCAC
Ehmt2	AGACAGCCCGTGGGTGAA	GCGGTCAATCTTGGGAGC
Hmga1	CCCCAGCTCGGATGTGAC	GCAAATGCGGATCTGAAACC
Gapdh	ACCACAGTCCATGCCATCAC	TCCACCACCCTGTTGCTGTA
